# Biomarkers of Prostatic Cancer: An Attempt to Categorize Patients into Prostatic Carcinoma, Benign Prostatic Hyperplasia, or Prostatitis Based on Serum Prostate Specific Antigen, Prostatic Acid Phosphatase, Calcium, and Phosphorus

**DOI:** 10.1155/2017/5687212

**Published:** 2017-01-12

**Authors:** Shahana Sarwar, Mohammed Abdul Majid Adil, Parveen Nyamath, Mohammed Ishaq

**Affiliations:** ^1^Department of Biochemistry, Owaisi Group of Hospitals, DCMS, Hyderabad, Telangana, India; ^2^Department of Urology, Owaisi Group of Hospitals, DCMS, Hyderabad, Telangana, India; ^3^Salar-e-Millat Research Centre, PEH, DCMS, Hyderabad, Telangana, India

## Abstract

Prostatitis, BPH, and P.Ca are the most frequent pathologies of the prostate gland that are responsible for morbidity in men. Raised levels of PSA are seen in different pathological conditions involving the prostate. PAP levels are altered in inflammatory or infectious or abnormal growth of the prostate tissue. Serum calcium and phosphorus levels were also found to be altered in prostate cancer and BPH. The present study was carried out to study the levels of PSA, PAP, calcium, and phosphorus in serum of patients with Prostatitis, BPH, or P.Ca and also to evaluate the relationship between them. Males in the age group of 50–85 years with LUTS disease symptoms and with PSA levels more than 4 ng/mL were included. A total of 114 patients were analyzed including 30 controls. Prostatitis in 35.7% of cases, BPH in 35.7% of the cases, and P.Ca in 28.57% of the cases were observed. Thus, the nonmalignant cases constitute a majority. PSA, a marker specific for prostatic conditions, was significantly high in all the diseases compared to controls. A rise in serum PSA and PAP indicates prostatitis or, in combination with these two tests, decreased serum calcium shows advanced disease.

## 1. Introduction

The diseases associated with prostate are prostatitis, benign prostatic hyperplasia (BPH), and prostate cancer (P.Ca) which leads to several metabolic disturbances. P.Ca is the second dreadful cancer in the world, common among men in US. Annually more than 2,30,000 men are diagnosed and approximately 30,000 die from it. African American men have high incidence of P.Ca and 60% more likely to develop when compared to Caucasian. It affects the young men with positive family history of P.Ca when compared to individuals without history. Men above 45 years are prone to BPH, a universal phenomenon which increases with age. Various factors are responsible for the prostate diseases in men especially elders [1, 2]. Screenings for P.Ca include serum PSA and digital rectal examination (DRE); a biopsy is required to diagnose P.Ca. The histopathological diagnosis of prostate cancer will only confirm the final diagnosis in 67% of cases whereas 33% are diagnosed with the other modalities [3].

Early detection and treatment in asymptomatic men may improve the mortality rate and the quality of life. Screening for markers such as prostate specific antigen (PSA) and prostate acid phosphatase (PAP) resulted in detection and treatment of the disease at an earlier stage.

Men of 50 years of age or above without any family history of cancer and those at 40 years of age with family history must undergo digital rectal examination (DRE) and PSA levels should be checked annually as recommended by of American Urological Association (AUA) and Food and Drug Administration (FDA).

The screening of PSA is not a common practice in India. The patients visiting department of urology of a hospital with complaints of lower urinary tract symptoms (LUTS) are checked for their PSA levels. Various studies reported that men with LUTS have the same risk of having P.Ca as asymptomatic men of the same age and have an increased risk of unnecessary biopsy if the threshold is taken as the same as that in case of the symptomatic men [4–6].

Several studies reported that markers such as PSA and PAP were used for confirming and monitoring P.Ca. PSA is one of the organ specific tumor markers produced by prostatic tissue [7]. In patients with BPH, PSA levels can increase 2-3 times the normal. The major limitation for using PSA, as a screening prostate cancer biomarker, is that majority of the men suffer from BPH and prostatitis as they become old, which increases their serum PSA levels. Therefore, PSA alone cannot be used as a biomarker for cancer detection. The elevated levels of PSA do not indicate cancer but the higher the PSA level, the more the chance of having cancer.

PAP is a tumor marker produced by the lysosomes of the prostate's epithelial cells [8]. The levels of PAP are high in 60% of men with P.Ca with metastases. However, the level of PAP in the serum is normal or slightly high when the carcinoma remains localized in the prostate gland and is elevated in some benign condition such as BPH and osteoporosis.

The association between high levels of serum calcium and risk of P.Ca has been demonstrated by various investigators [9]. The high level of calcium in serum is due to decrease in apoptosis and an increase in proliferation of P.Ca cells responsible for growth and metastasis. The increase in serum calcium or any factor that leads to it would increase the possibility for terminal P.Ca as has been reported by several researchers. Thus, analysis of calcium in serum may be used as a promising prospective biomarker for screening for P.Ca.

Several studies have reported a positive association between phosphorus intake and P.Ca [10, 11]. A recent study by Wilson et al. showed that calcium and phosphorus have independent effects at different time periods between exposure and diagnosis of P.Ca [12].

There is a need to use combination of markers and tests which can save the patient from unnecessary biopsies.

The present study was carried out to categorize the patients into prostatic carcinoma, benign prostatic hyperplasia, or prostatitis based on serum prostate specific antigen, prostatic acid phosphatase, calcium and phosphorus levels, and DRE and to evaluate the discriminating power of these in distinguishing controls and cases to reduce the risk of unnecessary biopsy. The diagnostic efficiency (DE) of these markers was confirmed by analyzing their sensitivity and specificity using receptor operative curves (ROC).

## 2. Methods

The study was approved by Institutional Ethical Committee. The informed consent was obtained from the patients. The patients with LUTS visiting the Department of Urology at Owaisi group of hospitals and research centre were enrolled in the study. A total of 114 subjects were included in the study. 84 patients served as cases whereas age and sex matched 30 individuals were selected from ENT, dental, and gastroenterology departments of the same hospital with complaints of disease other than LUTS and served as controls. From each department, 10 subjects were included.

### 2.1. Inclusion Criteria

Male patients in the age group of 50–85 years with LUTS and with PSA levels more than normal value (i.e. >4 ng/mL) were included. Age and sex matched persons without LUTS served as controls.

### 2.2. Exclusion Criteria

Patients with acute LUTS with fever and other symptoms, documented UTI, established cases of P.Ca, BPH and prostatitis, or history of previous biopsy or urological procedure were excluded.

The clinical diagnosis of prostatic disease was based on LUTS, elevated levels of PSA, DRE, TRUS, and TRUS guided biopsy, and histopathological examination of the biopsied tissue. The blood was collected at initial presentation in order to avoid any stimulation of the prostatic gland, which may increase PSA levels. Then, the patients were categorized into prostatitis, BPH, and P.Ca.

### 2.3. Sample Collection

10 mL of venous blood (fasting) was collected under aseptic conditions in the plain tubes. Serum was used for the analysis of PSA, PAP, calcium, and phosphorus.

### 2.4. Procedure

Serum PSA was measured using commercially available kits based on enzyme immunoassay for the quantitative determination by Pathozyme ELISA [13]. The estimation of PAP was done by Kinetic method [14], calcium by O-cresolphthalein complexone method and End Point Assay [15], and the level of phosphorus was done by UV-End point method [16]. The ROC was plotted by Youden Index, to calculate the best cutoff value.

## 3. Results

The age of the subjects were given in the age range of 50–85 years (Table 1).

The mean values for PSA, PAP, and calcium are significantly higher in cases than controls (Table 2). The comparison of PSA and PAP levels in between the groups revealed that the levels are significantly higher in prostatitis as compared to BPH and controls. The level of PAP was significantly higher in prostatic carcinoma as compared to prostatitis. The serum calcium level was higher in BPH group when compared to prostate carcinoma. The phosphorus level was significantly more in BPH than controls.

**Figure 1:**
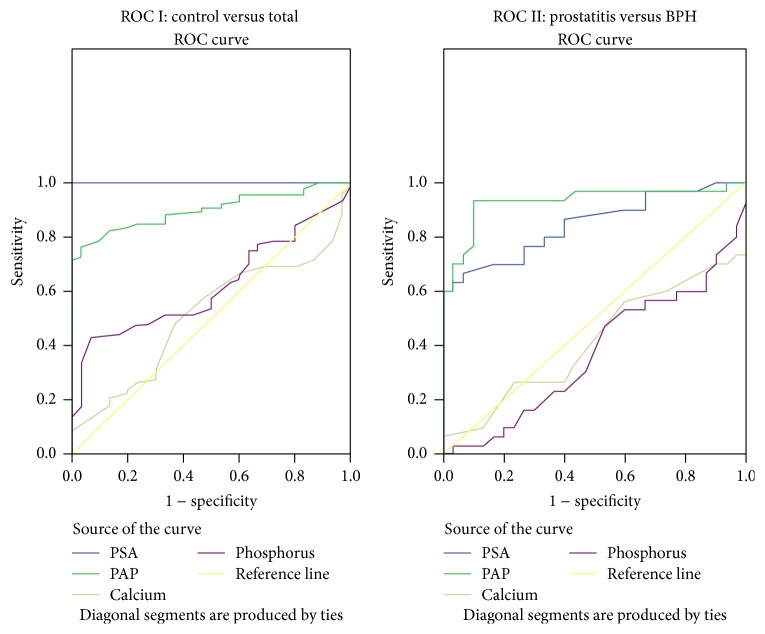
Receptor operative curves.

**Figure 2:**
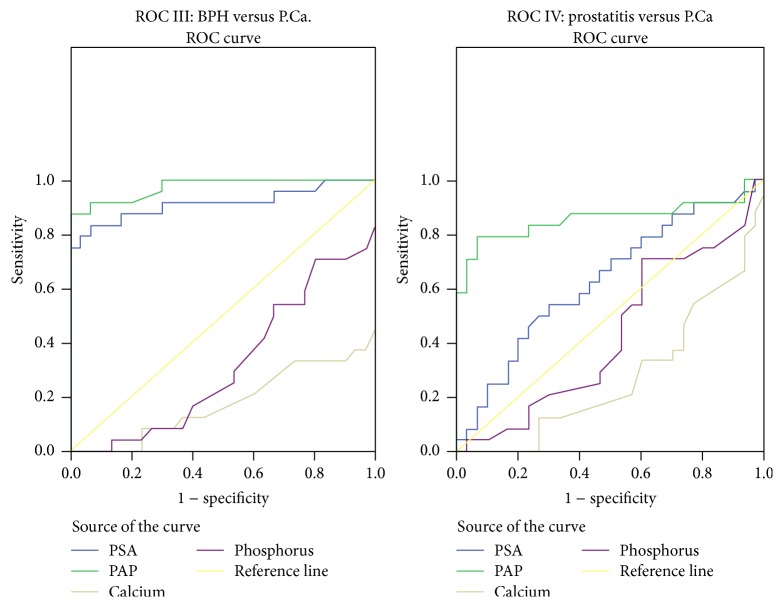
Receptor operative curves.

In order to assess the maximum sensitivity, specificity, and DE of various parameters in identifying abnormality, the best cutoff values (BCV) were calculated using ROC analysis (Figures 1 and 2, Tables 3 and 4). The analysis of prostate gland in patients of various groups based on digital rectal examination (DRE) was given in Table 5. The PSA levels and staging of prostate carcinoma patients in different age groups were given in Table 6.

 In cases and controls,

PSA, at bcv 4.75 ng/mL, showed 100% sensitivity and specificity,
PAP, at bcv 4.05 IU/L, showed 76% sensitivity and 96% specificity,
serum phosphorus 4.35 mg/dL showed specificity of 93%.

In prostatitis and BPH,

PSA, at bcv 16.75 ng/mL, showed 70% sensitivity and 83.3% specificity,
PAP, at bcv 6.35 IU/L, showed 90% sensitivity and specificity,
serum calcium 10.6 mg/dL showed 100% specificity,
phosphorus 6.65 mg/dL showed 99.9% sensitivity and 96.7% specificity.

In BPH and P.Ca,

PSA, at bcv 22.5 ng/mL, showed 99.27% sensitivity and 96.7% specificity,
PAP, at bcv 10.5 IU/L, showed 87.5% sensitivity and 100% specificity,
serum calcium, 5.8 mg/dL, showed 100% sensitivity.

In prostatitis and P.Ca,

PSA, at bcv 36.3 ng/mL, showed 70.8% sensitivity and 50% specificity,
PAP, at bcv 19.5 IU/L, showed 79.2% sensitivity and 93% specificity,
serum calcium 5.8 mg/dL showed 100% sensitivity and no specificity.

## 4. Discussion

Prostate cancer, prostatitis, and BPH are the most frequent pathologies of the prostate gland, whose management strategies are diametrically different. P.Ca is the most frequently diagnosed malignancy in men and the second leading cause of cancer deaths especially in Western countries [1]. There is lack of epidemiological data on the exact prevalence of this disease in India due to lack of proper screening and underrecorded incidence of P.Ca. On the other hand, BPH and prostatitis are the other two most frequent pathologies of prostate gland that clinically/symptomatically mimic prostate cancer. P.Ca is notoriously difficult to treat, which makes its early detection a priority. There is an urgent need for appropriate diagnostic and prognostic markers to detect P.Ca and to differentiate it from other pathologies of prostate gland. The present study was undertaken to assess the levels of PSA, PAP, calcium, and phosphorus in different pathologies of prostate gland, in an attempt to use a combination of markers to differentiate the above conditions to avoid the use of unnecessary biopsies. Our observations revealed that PSA was significantly high in cases than controls (*p* < 0.001). These findings correlated with the studies by Jung et al. and Anim et al. confirming the increased levels of PSA in different pathologies of prostate gland [17, 18].

The discovery of PSA as a biomarker and demonstration of its utility in early diagnosis and monitoring of P.Ca date back to the early 1980s. However, the use of only PSA as an initial diagnostic tool has become controversial over the past decade due to its increased levels in other pathologies of prostate. This view is supported by our findings of significantly higher increase in PSA in prostatitis and prostate cancer compared to controls and BPH. The above marker, though it remains an early signal for the pathologies of prostate, may lead to overdiagnosis which in turn would result in over and aggressive treatment strategies. PAP was significantly increased in cases compared to controls (*p* < 0.001). Our findings were in agreement with the earlier studies by Taira et al. [19] and Nguyen et al. [20] thereby confirming the increased levels of PAP in different pathologies of prostate gland.

PAP emerged as the world's first clinically useful tumor marker in the 1940s and 1950s. With the introduction of the PSA test in the 1980s, which performed significantly better than PAP in terms of screening and monitoring response to treatment, PAP fell into disfavor. The recent studies have identified PAP as a significant prognostic factor for patients with intermediate and high-risk prostate cancer. PAP was known to have a low sensitivity for diagnosing new disease. In a study it was found that PAP has low sensitivity compared to PSA [21]. Since the goal of P.Ca screening is to identify early-stage treatable disease, PAP was rightly dropped as a screening tool. Several studies suggest that PAP could play a role in determining which early-stage patients are likely to benefit from more aggressive adjuvant therapy [21]. This view is supported by our findings of significantly higher increase in PAP in prostatitis and P.Ca compared to controls and BPH; in addition, we also observed significant increase in serum PAP in prostate cancer compared to prostatitis.

Moreover, it was observed that, at cutoff value of 4.75 ng/mL, PSA is indicative of prostate gland pathology at a sensitivity and specificity of 100% and DE of 100% which may lead to differentiating any pathology. However, the DE in discriminating prostatitis from BPH is 76.6%, between P.Ca and BPH is 90.7%, and between prostatitis and P.Ca is only 59.2%. Thus, the discriminating or diagnosing power of PSA into the type of pathology is limited. Our findings were in accordance with those of Yaman et al. and Simardi et al. who related the PSA elevation to pathologies of prostate tissues [22, 23]. It was also observed that, at a cutoff value of 4.05 IU/L, PAP is indicative of prostate gland pathology at a sensitivity of 76.2% and specificity of 96.7% in discriminating cases from controls and a DE of 82.4%. Thus, PAP was found to have a better discriminating power into the type of pathology. Its DE to differentiate between prostatitis and BPH is 90%, between BPH and P.Ca is 94.4%, and prostatitis from P.Ca is 85.5%.

A rise in PSA and PAP, in combination, indicates either prostatitis or P.Ca and rules out BPH. In combination to these two, a decrease in serum calcium is indicative of advanced disease in prostate cancer. This is in accordance with the findings of Kukreja et al. which show a significant decrease in P.Ca [9]. According to them, in patients with solid tumors like prostate, breast, or lung cancer, hypocalcaemia develops due to extensive osteoblastic metastases. Raskin et al. performed a retrospective analysis of serum calcium levels in patients with metastatic bone disease and reported a 33% prevalence of hypocalcaemia in patients with prostate cancer [24]. This is also in accordance with study by Szentirmai et al. which revealed that few patients with prostate cancer and bone metastases have a low serum calcium concentration, and some have severe hypocalcaemia [25]. Avid calcium uptake by osteoblastic bone metastases was postulated more than 30 years ago by Ludwig [26]. The hypocalcaemia in these patients may be most likely on the basis of extensive accretion of calcium into bones. Serum total calcium was significantly lower in patients with bone metastasis than those without. In this case, hypocalcaemia can be explained on the basis of hypoalbuminemia or renal failure. Therefore, apparent hypocalcaemia based on total calcium measurement is common in patients with P.Ca [9]. Men whose blood calcium levels fall at the high end of the normal range are more likely to develop 2.5 times fatal P.Ca when compared to men with lower levels, according to a recent study. If confirmed by other studies, the findings could have important implications for the prevention and treatment of P.Ca [27].

It has been known for many years that hypocalcaemia can occur in patients with osteoblastic metastases from prostate cancer. Ludwig postulated the following sequence: osteoblastic metastases cause increased deposition of calcium and phosphate in bone, tending to decrease serum concentrations of both ions [27]. P.Ca, BPH, and prostatitis are common prostatic clinical conditions whose management strategies are diametrically different. The final areas in differentiating these conditions are biopsy. Biopsies (8–10 needle core) have its own complications and side effects.

Though PSA has emerged as marker for prostatic conditions like P.Ca and BPH in the past decade, there has been an increasing realization by both biochemists and urologists, to have a better single marker or a combination of biochemical tests to differentiate the above conditions, to avoid frequency and unnecessary biopsies in nonmalignant conditions.

PSA is a marker specific for prostatic conditions and shown to be significant in all disease compared to controls. So, a rise in serum PSA and PAP indicates prostatitis or, in combination with these two tests, decreased serum calcium shows advanced disease.

## Figures and Tables

**Table 1:** The number of patients in each group along with their age.

Group	Age (years)				
	50–59	60–69	70–79	80+	Total
Controls	7	9	12	2	30
Prostatitis	13	11	5	1	30
BPH	8	11	6	5	30
P.Ca	3	8	9	4	24

This table shows the age range of patients and controls included in the study. A total of 114 patients were included in the study. The individuals were grouped into 4 groups based on the age range which was from 50 to 85 years.

**Table 2:** The table shows the mean levels of various biomarkers in different groups.

Group	PSA (ng/mL)	PAP (IU/L)	Calcium (mg/dL)	Phosphorus (mg/dL)
Control	2.27 ± 1.06	2.5 ± 1.12	8.94 ± 0.74	3.41 ± 0.91
Prostatitis	52 ± 6.45	11.4 ± 2.34	9.08 ± 0.93	3.77 ± 1.1
BPH	11.15 ± 2.23	3.9 ± 1.34	9.23 ± 0.78	4.85 ± 1.8
Prostate Carcinoma	70.64 ± 6.54	25.1 ± 3.4	8.45 ± 0.85	3.5 ± 1.98

The table showed the levels of biochemical parameters in different groups. The levels of PSA and PAP were higher in P.Ca group as compared to the others.

**Table 3:** The sensitivity and specificity of various biomarkers in different groups are given in the table.

Parameter		Control versus total	Prostatitis versus BPH	BPH versus P.Ca	Prostatitis versus P.Ca
PSA (ng/mL)	Best cutoff	4.75	16.75	22.5	36.3
	Sensitivity	100	70	99.2	70.8
	Specificity	100	83.3	96.2	50

PAP (IU/L)	Best cutoff	4.05	6.35	10.5	19.5
	Sensitivity	76.2	90	87.5	79.2
	Specificity	96.7	90	100	93

Calcium (mg/dL)	Best cutoff	8.95	10.6	5.8	5.8
	Sensitivity	47.6	6.7	100	100
	Specificity	63.3	100	0	0

Phosphorus (mg/dL)	Best cutoff	4.35	6.65	5.55	3.1
	Sensitivity	42.9	99.9	4.25	70.8
	Specificity	93.3	96.3	86.7	40

The table shows the sensitivity and specificity pattern of PSA and PAP in different groups. At BCV 4.75 ng/mL, the PSA showed 100% sensitivity and specificity whereas at BCV 4.05 IU/L PAP showed 76.2% sensitivity and 96.7% specificity. BCV at 5.8 mg/dL calcium showed 100% sensitivity in BPH versus P.Ca and prostatitis versus P.Ca.

**Table 4:** The percentage of diagnostic efficiency (DE).

Parameter	Control versus total	Prostatitis versus BPH	BPH versus P.Ca
PSA	100%	76.6%	59.2%
PAP	82.4%	90%	83.35

The table showed the percentage of diagnostic efficiency of PSA and PAP in cases and controls. Serum PSA showed 100% DE in total cases and control whereas PAP showed only 82.4%. PSA showed DE of 76.6% and 59.2% in prostatitis versus BPH and BPH versus P.Ca, respectively, whereas DE of PAP was 90% and 83.3% in the above groups.

**Table 5:** The analysis of prostate gland in patients of various groups based on digital rectal examination (DRE).

Group	Number of patients	DRE details
Control (*n* = 30)	15	Normal, firm, nontender prostate with maintained landmarks
	13	Grade I-II, nontenderness and maintained land marks
	2	Grade III-IV, prostatomegaly

Prostatitis (*n* = 30)	16	Grade I-II prostatic enlargement with mild to moderate tenderness with maintained land marks
	7	Grade I-II with no tenderness with maintained landmarks
	4	Grade III-IV with mild to moderate tenderness and normal landmarks, firm consistency
	3	Grade III-IV with severe tenderness and soft prostate consistency

BPH (*n* = 30)	12	Grade I-II
	9	Grade III
	9	Grade IV, firm, nontender with maintained landmarks (median groove, etc.), no induration

**Table 6:** The PSA levels and staging of prostate carcinoma patients.

Age (years)	Number of patients	PSA (ng/mL)	TNM staging	Stage	Gleason score
50–59	3	66.75	T_1_N_0_ M_0_	I-II	3 + 3 = 6
60–69	8	68.82	T_1_N_1_ M_0_	II-III	4 + 4 = 8
70–79	4	78.45	T_3_N_1_ M_1_	III-IV	5 + 4 = 9
	5	76.62	T_3_N_X_M_1_	III-IV	3 + 4 = 7
80+	4	70.46	T_2_N_0_ M_1_	I-II	3 + 4 = 7

The table showed the TNM (tumor, lymph nodes, and metastases) staging and Gleason score in patients of various age groups. The level of PSA was also included.
